# Associations of *TIMP-3* Genetic Polymorphisms with *EGFR* Statuses and Cancer Clinicopathologic Development in Lung Adenocarcinoma Patients

**DOI:** 10.3390/ijms21218023

**Published:** 2020-10-28

**Authors:** Jer-Hwa Chang, Tsung-Ching Lai, Po-Jen Yang, Pei-Chun Shih, Yi-Chieh Yang, Kai-Ling Lee, Tu-Chen Liu, Thomas Chang-Yao Tsao, Shun-Fa Yang, Ming-Hsien Chien

**Affiliations:** 1 School of Respiratory Therapy, College of Medicine, Taipei Medical University, Taipei 11031, Taiwan; m102094030@tmu.edu.tw; 2 Division of Pulmonary Medicine, Department of Internal Medicine, Wan Fang Hospital, Taipei Medical University, Taipei 116, Taiwan; 109053@w.tmu.edu.tw; 3 Pulmonary Research Center, Wan Fang Hospital, Taipei Medical University, Taipei 116, Taiwan; 4 School of Medicine, Chung Shan Medical University, Taichung 40201, Taiwan; cshy1030@csh.org.tw (P.-J.Y.); his885889@gmail.com (T.C.-Y.T.); 5 Department of Family and Community Medicine, Chung Shan Medical University Hospital, Taichung 40201, Taiwan; 6 Department of Laboratory Medicine, National Taiwan University Hospital, Taipei 100225, Taiwan; peichun1976@gmail.com; 7 Graduate Institute of Clinical Medicine, College of Medicine, Taipei Medical University, Taipei 11031, Taiwan; rafiyang@tmu.edu.tw (Y.-C.Y.); d118107005@tmu.edu.tw (K.-L.L.); 8 Department of Medical Research, Tungs’ Taichung MetroHarbor Hospital, Taichung 435, Taiwan; 9 Division of Pulmonary Medicine, Department of Internal Medicine, Taipei Medical University Hospital, Taipei 110301, Taiwan; 10 Department of Chest Medicine, Cheng-Ching General Hospital, Taichung 407, Taiwan; liou.dj@gmail.com; 11 Division of Chest, Department of Internal Medicine, Chung Shan Medical University Hospital, Taichung 40201, Taiwan; 12 Institute of Medicine, Chung Shan Medical University, Taichung 40201, Taiwan; 13 Department of Medical Research, Chung Shan Medical University Hospital, Taichung 40201, Taiwan; 14 TMU Research Center of Cancer Translational Medicine, Taipei Medical University, Taipei 11031, Taiwan; 15 Traditional Herbal Medicine Research Center, Taipei Medical University Hospital, Taipei 11031, Taiwan

**Keywords:** tissue inhibitor of metalloproteinases-3, polymorphism, epidermal growth factor receptor, mutation, lung adenocarcinoma

## Abstract

Lung adenocarcinoma (LADC) is a major subtype of lung cancer, particularly among populations of East Asia. The epidermal growth factor receptor (EGFR) is the most frequently mutated oncogene promoting LADC progression and can serve as a therapeutic target in LADC. The tissue inhibitor of metalloproteinases (TIMP)-3 is a major regulator of extracellular matrix turnover via targeting of matrix metalloproteinases (MMPs), and thus, plays a critical role in tumor development and progression. The purpose of this study was to investigate potential associations among TIMP-3 genetic polymorphisms, EGFR statuses, and cancer clinicopathologic development in patients with LADC. In this study, 277 LADC patients with different EGFR statuses were recruited to dissect the allelic discrimination of TIMP-3 -1296 T>C (rs9619311), TIMP3 249T>C (rs9862), and TIMP3 261C>T (rs11547635) polymorphisms using a TaqMan allelic discrimination assay. Our data showed that compared to those LADC patients with wild-type CC homozygotes of TIMP-3 rs9862, patients harboring TT homozygotes of rs9862 were at a higher risk of developing mutant EGFR (adjusted odds ratio (AOR) = 2.530; 95% confidence interval (CI): 1.230–5.205; *p* = 0.012), particularly the EGFR L858R point mutation (AOR = 2.975; 95% CI: 1.182–7.488; *p* = 0.021). Moreover, we observed that TIMP-3 TT homozygotes of rs9862 were correlated with the incidence of EGFR mutations in patients with a smoking habit (*p* = 0.045). Within male patients harboring a mutant EGFR, TIMP-3 rs9862 T (CT+TT) allele carriers were at higher risk of developing an advanced stage (*p* = 0.025) and lymph node metastasis (*p* = 0.043). Further analyses of clinical datasets revealed correlations of TIMP-3 expression with a favorable prognosis in patients with LADC. In conclusion, the data suggest that TIMP-3 rs9862 polymorphisms may contribute to identify subgroups of lung cancer patients at high risk for tumor progression, among carriers of LADC-bearing mutant EGFR.

## 1. Introduction

Lung cancer is the most prevalent cancer and is responsible for the top three mortality among various cancers globally [1]. About 90% of patients have non-small-cell lung cancer (NSCLC), and the remainder have small-cell lung cancer (SCLC). Lung adenocarcinoma (LADC) is the major subtype of NSCLC, accounting for about 40% of all lung cancer cases, and squamous cell carcinoma (SCC) is the second most common type. The epidermal growth factor receptor (EGFR) is a surface tyrosine kinase (TK) receptor, and its mutations are the prevalent oncogenic mutation observed in LADC, particularly among populations of East Asia, females, and non-smokers [2,3,4]. Most of these mutations exist within the catalytic kinase domain, which increases EGFR activity. The two most common somatic mutations, an exon 19 in-frame deletion and a point mutation L858R at exon 21, account for ~85% of all EGFR mutations in NSCLC, particularly in LADC [5,6,7]. Unregulated activity of the EGFR mediates proliferation, invasion, migration, and angiogenesis, which promote the progression of tumor cells [2]. Therefore, recent studies showed that LADC patients harboring somatic mutations of the EGFR TK domain are suitable for treatment with EGFR TK inhibitors (TKIs) [8].

Tissue inhibitor of metalloproteinase (TIMP)-3 belongs to the TIMP family, comprising TIMP-1~4, which are endogenous regulators of matrix metalloproteinases (MMPs) and important for maintaining the adjacent extracellular matrix (ECM). TIMP-3 is known to have the broadest inhibition spectrum against activities of MMPs, such as the families of a disintegrin and MMP (ADAM), and ADAM with thrombospondin motifs (ADAMTS) [9]. Increased activities of MMPs are usually associated with invasion and metastasis of LADC cells [10,11]. Therefore, downregulation of TIMP-3 by inducing histone H3 lysine 4 (H3K4) demethylation, promoter methylation, or a decrease in messenger (m)RNA stability was reported to be correlated with the invasive ability of NSCLC cells [12,13,14,15]. TIMP-3 expression is associated with favorable prognoses in various cancers such as colorectal cancer, liver cancer, and NSCLC [16,17,18]. In addition to MMPs, TIMP-3 was reported to block the binding of Vascular endothelial growth factor (VEGF) to VEGF receptor (VEGFR)-2 and inhibit downstream signaling and angiogenesis [19]. TIMP-3, through its inhibition of MMPs, was shown to inhibit EGFR signaling in cardiomyocytes and bronchial epithelial cells [20,21].

Genetic variations such as single-nucleotide polymorphisms (SNPs) can alter expressions of coding genes that are associated with the risk of developing cancer. Previously, cancer-related SNPs and somatic mutations were usually analyzed separately. Recently, increasing numbers of reports indicate the interplay between genetic variants and somatic mutations in cancer development. For example, SNP rs17000526 in apolipoprotein-B mRNA-editing catalytic polypeptide-like (APOBEC)3 was reported to alter the expression of APOBEC3A/APOBEC3B, which affects somatic APOBEC-signature mutations [22]. In lung cancer, a cancer-related SNP, telomerase reverse transcriptase (TERT) rs2736100, was shown to be associated with susceptibility to EGFR mutations in NSCLC [23]. Some cancer-related SNPs at five loci—TERT, butyrophilin-like 2, TP63, bromodomain PHD finger transcription factor, and high-mobility group box protein 1—showed significant effects on EGFR-mutated LADC [24,25]. To date, whether polymorphisms of TIMP-3 play any role in the risk of somatic mutations of the EGFR in LADC remains unknown, especially in Asian populations.

Thus, we performed a case–control study in a Taiwanese population to investigate correlations of TIMP-3 SNPs with EGFR mutation statuses and clinical features in LADC. Three SNPs across TIMP-3 (rs9619311, rs9862, and rs11547635) were examined in this study. The rs9619311 polymorphism locus located in the promoter region was reported to play a role in susceptibility to hepatocellular carcinoma [26]. The other two SNPs (rs9862 and rs11547635) are located in coding exon regions, and rs9862 was reported to be associated with prognosis of adenocarcinomas of the gastroesophageal junction [27].

## 2. Results

### 2.1. General Characteristics of LADC Patients Harboring the Wild-Type (WT) or Mutant EGFR

There were 277 patients with LADC in this study. Their demographic and clinical characteristics are described in Table 1. Samples were divided into two groups: the WT EGFR and mutant EGFR. There were 109 patients with the WT EGFR (65 males and 44 females; mean age of 65.45 ± 13.34 years) and 168 patients with the mutant EGFR (60 males and 108 females; mean age of 65.69 ± 13.58 years). Significantly more females had the mutant EGFR than males. Regarding cigarette smoking status, EGFR TK domain mutations were significantly more frequent in those who had never smoked than in those who had (77.4% vs. 22.6%). Overall, the demographic characteristics of our recruited EGFR-mutant LADC subjects were consistent with Asian LADC patients as previously reported [3]. Regarding the clinical features of these patients, an EGFR mutation was only correlated with well/moderately differentiated cells, but not with the clinical stage (I+II vs. III+IV), T status (T1+T2 vs. T3+T4), N status (negative vs. positive), or M status (negative vs. positive).

### 2.2. Associations between TIMP-3 Candidate SNPs (rs9619311, rs9862, and rs11547635) and EGFR Mutations in LADC Patients with or without Cigarette Consumption

To examine possible associations of TIMP-3 SNPs with the risk of developing EGFR mutations, the genotype frequencies of three SNPs (rs9619311 (promoter region, -1296T>C), rs9862 (coding exon 3, 249T>C), rs11547635 (coding exon 3, 261C>T)) were first investigated in all LADC patients harboring the WT or mutant EGFR. The highest frequencies of these three TIMP-3 SNPs were homozygous for TT, CC, and CC, respectively, in both WT and mutant EGFR groups. After adjusting for age, gender, and cigarette smoking, rs9619311 and rs11547635 SNPs showed no significant correlation with the risk of developing EGFR mutations (Table 2). In contrast, the TT and CT+TT genotypes of TIMP-3 rs9862 were significantly associated with an increased ratio of EGFR mutations (AOR: 2.530, 95% CI: 1.230–5.205, *p* = 0.012; AOR: 1.748, 95% CI: 1.002–3.048, *p* = 0.049, respectively). Moreover, cigarette consumption was reported to impact the EGFR mutation rate in a Taiwanese LADC population [28]. We divided our recruited LADC patients into smoking and non-smoking groups and observed that smokers with the TT genotype of rs9862 had a 3.497-fold risk (95% CI: 1.027–11.904, *p* = 0.045) of developing EGFR mutations (Table 3).

Next, we divided EGFR mutations into two major mutant types, L858R (*n* = 78) and exon 19 in-frame deletion (*n* = 81) groups, and further investigated the associations of these two mutant types of the EGFR gene with TIMP-3 genotypes. As shown in Table 4, rs9619311 and rs11547635 still exhibited no correlation with either hotspot mutation of the EGFR. For rs9862 polymorphisms, the TT genotype exhibited a significantly higher 2.975-fold frequency of having an EGFR L858R mutation compared to the corresponding WT homozygotes, but there was no significant association demonstrated between rs9862 variant types and the EGFR exon 19 in-frame deletion (Table 4).

### 2.3. Correlations between Polymorphic Genotypes of TIMP-3 and Clinicopathological Characteristics of LADC Patients of Different Genders with the WT or Mutant EGFR

Because rs9862 is associated with the incidence of EGFR mutations in LADC patients with a smoking habit, we further analyzed correlations of variant genotypes of TIMP-3 rs9862 with the clinical characteristics in our recruited cohort. An association was reported between smoking behaviors and gender, particularly among Asian populations, which indicated that the smoking rate was significantly higher in males than in females [28]. We divided our recruited LADC patients into male and female groups and observed a significant association of rs9862 variants (CT+TT) with advanced stage tumors (stage III/IV; OR: 2.571; 95% CI: 1.069–6.187, *p* = 0.032) in the male population with the WT or mutant EGFR (Table 5). We next divided male LADC patients into WT and mutant EGFR groups and observed that 60 patients who harbored at least one polymorphic T allele of TIMP-3 rs9862 had enhanced risks of developing advanced stage tumors (stage III/IV; OR: 4.047; 95% CI: 1.136–14.411, *p* = 0.025) and lymph node metastasis (OR: 3.500; 95% CI: 1.003–12.216, *p* = 0.043) (Table 6).

## 3. Discussion

In our recruited cohort, LADC patients with an EGFR mutation were more frequent in non-smokers and females, and also had better cell differentiation, compared to LADC patients in the WT EGFR group. Compared to two East Asian cohorts and a global cohort, the characteristics of our recruited cohort were consistent with previous reports indicating that being female, Asian, and a non-smoker were the main characteristics of patients with a mutant EGFR [29,30,31]. In this study, we provided a novel finding that LADC patients with a smoking history harboring TIMP-3 rs9862 TT homozygotes had a significantly higher incidence of developing an EGFR mutation. Moreover, the male population harboring a mutant EGFR and T allele rs9862 polymorphism had a higher risk of developing advanced stage tumors and lymph node metastasis.

**Figure 1:**
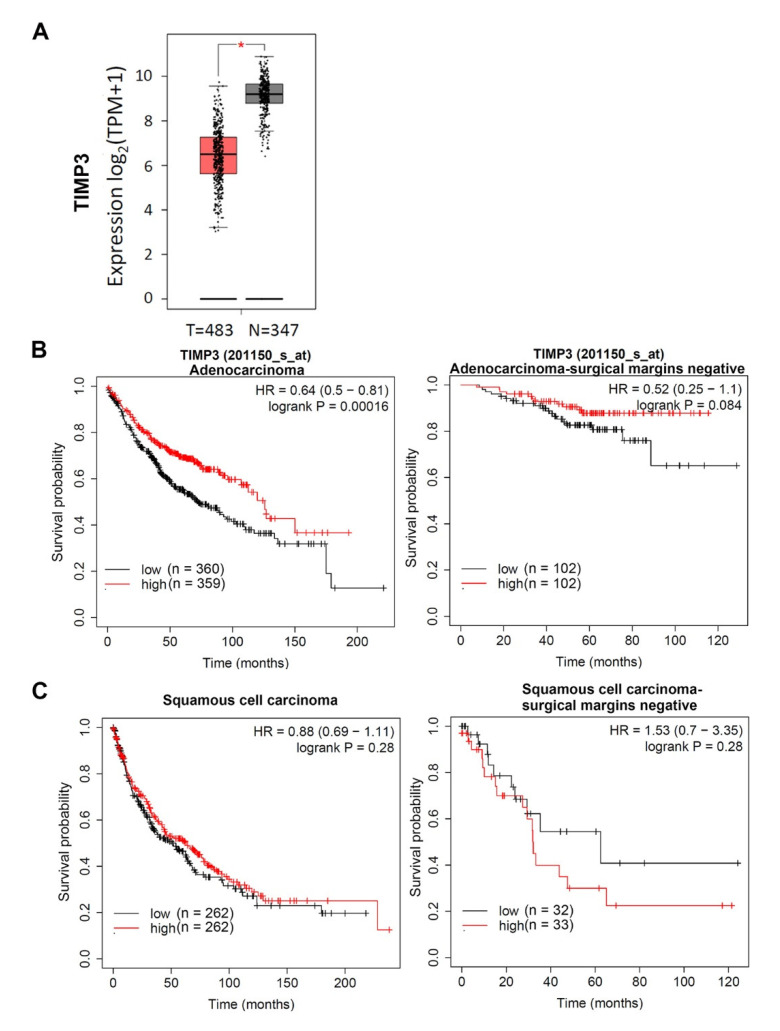
Clinical significance of tissue inhibitor of metalloproteinases (TIMP)-3 in lung adenocarcinoma (LADC). (**A**) Gene expressions of TIMP-3 between tumor and normal samples in the combined data of The Cancer Genome Atlas (TCGA)-LADC and Genotype-Tissue Expression (GTEx) cohorts. (**B** and **C**) Correlation between TIMP-3 expression and the overall survival rate in LADC and squamous cell carcinomas using the Kaplan–Meier plotter database. Gene expressions were dichotomized into high and low values using the median as a cutoff. HR—hazard ratio.

In the past decade, several reports showed that TIMP-3 acts as a tumor suppressor in cancer cells. In cell-based experiments, TIMP-3 was reported to suppress cell migration and invasion by inhibiting the activity of MMPs and ADAMs in many cancers such as NSCLC, thyroid cancer, melanomas, liver cancer, and colorectal cancer [12,32,33,34]. Epithelial–mesenchymal transition (EMT) is an important event in promoting cancer metastasis [35,36,37] and determining the sensitivity of NSCLC to EGFR TKI [38]. Overexpression of TIMP-3 was shown to suppress EMT-induced metastasis in oral cancer [39]. The crosstalk between cancer cells-derived fibronectin and stromal fibroblast plays a critical role for tumor metastasis via inducing MMP-2 upregulation [40,41,42]. Upregulation of the MMP-2 in the fibroblasts neighboring the tumor was observed in about 50% of the NSCLC patients, enabling malignant transformation by ECM degradation and the creation of a suitable microenvironment for vessel growth [43]. TIMP-3 was reported to form a terminal complex to inhibit MMP-2 activation and inhibit the fibroblast-induced angiogenic phenotype of endothelial cells [44]. Moreover, TIMP-3 restoration induces various types of cancer cells’ apoptosis and inhibits cell proliferation [34,45]. 1,25-dihydroxyvitamin D, an anticancer agent [46], was shown to inhibit cell proliferation and invasion of endometrial cancer cells via upregulating TIMP-3 [47]. In clinical studies, decreased expression of TIMP-3 was correlated with a poor prognosis of patients with colorectal and liver cancer [16,18]. Actually, we also observed that significantly higher TIMP-3 transcripts were observed in normal tissues compared to LADC tumors (*p* < 0.005) from The Cancer Genome Atlas (TCGA) and Genotype-Tissue Expression (GTEx) datasets (Figure 1A). Furthermore, from the Kaplan–Meier (KM) plotter database, we observed that high levels of TIMP-3 were correlated with favorable prognostic outcomes in patients with LADC, even though patients had negative surgical margins (Figure 1B). In contrast, a favorable prognostic role of TIMP-3 was not observed in SCC (Figure 1C). In conclusion, TIMP-3 may be an LADC-specific prognostic factor of NSCLC, and downregulation of TIMP-3 is critical for cancer progression. In contrast to the tumor-suppressive role of TIMP-3, Kornfeld et al. reported that head and neck cancer patients expressing high levels of TIMP-3 mRNA had poor prognoses compared to those with low mRNA levels [48]. Epigenomic hypermethylation of the TIMP-3 promoter region was observed to reduce transcriptional activity in lung, kidney, brain, and oral cancers [12,39,49]. MicroRNA17 (miR17), miR20a, miR21, and miR181b can also reduce TIMP-3 expression in tumors [50,51,52]. SNPs are also genetic alterations, but no direct evidence has indicated that any SNP of TIMP-3 can influence its expression. Recently, among oral cancer patients with a betel quid chewing habit, we observed that subjects carrying the T allele rs9862 polymorphism had higher plasma levels of TIMP-3 compared to subjects carrying the CC genotype [53], suggesting that the CT or TT genotype might affect TIMP-3 expression in oral cancer. rs9862 is located in exon 3 within the TIMP-3 coding region. Although this SNP does not result in an amino acid change, rs9862 was reported to affect unidentified protein binding and may have a functional role by influencing gene expression to further alter the survival of patients with gastroesophageal junction adenocarcinomas [27]. The plasma levels of TIMP-3 and correlations of TIMP-3 expression with EGFR mutations in LADC patients carrying different rs9862 polymorphisms should be further addressed in future studies.

Our study observed that the rs9862 TT homozygous polymorphism was associated with a significantly higher proportion of patients developing EGFR mutations compared to the homozygous CC polymorphism in the smoking population, suggesting that the T allele of rs9862 might be a promoter of developing EGFR mutations in individuals who smoke. Cigarette smoking, a predominant factor for lung tumorigenesis, was reported to decrease TIMP-1, TIMP-2, or TIMP-3 expression in patients with heart disease or NSCLC [50,54]. Although EGFR mutations are commonly observed in NSCLC patients who were never smokers, approximately 40% of patients with EGFR mutations are former and current smokers [55]. The cumulative smoking dose was reported to be an important predictive and prognostic factor in LADC patients harboring mutant EGFR, and associated smoking-related gene signatures, such as point mutation of p53 and a high number of single-nucleotide variants, might affect outcomes after TKI treatment [56]. Actually, TIMP-3 was reported to be a biomarker associated with TKI resistance in cancer cells [57]. Whether the TIMP-3 rs9862 polymorphism can influence the outcome of LADC patients after TKI treatment needs further investigation.

## 4. Materials and Methods

### 4.1. Patient Specimens

We recruited 277 patients with LACD harboring different EGFR statuses from Taichung Cheng-Ching General Hospital (Taichung, Taiwan) in 2012–2015. Demographic data, lifestyle variables, and clinical characteristics comprising age, gender, cigarette smoking, stage, TNM status, and cell differentiation were all obtained from the medical records of each patient. The clinical stage of patients was judged according to the seventh edition of the *American Joint Committee on Cancer Staging Manual*. Informed consent was collected, and the study protocol was approved by the Institutional Review Board of Cheng-Ching General Hospital (no. HP120009; 22 September 2012).

### 4.2. DNA Extraction and EGFR Gene Sequencing from Tumor Tissues

The EGFR mutations in tissue specimens were detected by matrix-assisted laser desorption/ionization–time of flight mass spectrometry (MALDI–TOF MS) as described previously [58,59]. Briefly, DNAs were extracted from the paraffin-embedded tissues either by the QIAmp DNA Mini kit or the automated QIAsymphony extraction system with the QIAsymphony DNA kit (Qiagen, Valencia, CA), following the manufacturer’s protocol. MALDI–TOF MS was used to detect the genetic alterations of EGFR. The analysis of the results was performed according to the manufacturer’s protocol for the MassARRAY system (Agena Bioscience, San Diego, CA, USA).

### 4.3. Genomic TIMP3 SNPs Detected from Blood

Peripheral blood samples of study subjects were harvested in EDTA tubes, and genomic DNA was extracted with a QIAamp DNA blood mini kit (Qiagen). The three SNP (rs9619311 (assay ID: C_1840822_10), rs9862 (assay ID: C_3294861_10), and rs11547635 (assay ID: C_3294860_10)) genotypes of each sample were determined by using a TaqMan 5′-nuclease chemistry, including sequence-specific forward and reverse primers and two TaqMan minor groove binder probes with nonfluorescent quenchers. The TaqMan SNP Genotyping Assay used the ABI StepOnePlus^TM^ Real-Time PCR System (Applied Biosystems, Foster City, CA, USA). The allelic frequency was calculated with ABI SDS vers. 3.0 software.

### 4.4. Statistical Analysis

Analyses were calculated with SAS software (vers. 9.1, 2005; SAS Institute, Cary, NC, USA). Demographic characteristics between the wild-type (WT) and mutant type EGFR were calculated by a Mann–Whitney U-test. Adjusted odds ratios (AORs) and 95% confidence intervals (CIs) were assessed by multiple logistic regression models. A *p* value of <0.05 indicated a statistically significant difference.

## 5. Conclusions

It is well known that EGFR mutation is frequently observed in patients who are female, non-smokers, and diagnosed with LADC. However, in LADC patients harboring mutant EGFR, smokers and males still occupied 40% and 31%, respectively [55]. Among Taiwanese LADC patients with an EGFR mutation, male smokers exhibited the worst prognosis [28], and few SNP biomarkers are available for such population. In this study, we first identified that TIMP-3 rs9862 SNPs are associated with susceptibility to EGFR mutations and clinicopathologic development of LADC in smokers and males. TIMP-3 rs9862 SNPs might be a potential predictor of disease progression in this particular population.

## Figures and Tables

**Table 1:** Demographics and clinical characteristics of 277 lung adenocarcinoma patients with wild-type or mutant-type epidermal growth factor receptor (EGFR).

Subject Characteristic	Wild-Type (*N* = 109)	Mutation Type (*N* = 168)	*p* Value
**Age,**			
mean ± SD (years)	65.45 ± 13.34	65.69 ± 13.58	0.885
**Gender, *n* (%)**			
Male	65 (59.6%)	60 (35.7%)	<0.001
Female	44 (40.4%)	108 (64.3%)	
**Cigarette smoking, *n* (%)**			
Non-smoker	49 (45.0%)	130 (77.4%)	<0.001
Ever-smoker	60 (55.0%)	38 (22.6%)	
Stage, *n* (%)			
I+II	25 (22.9%)	47 (28.0%)	0.350
III+IV	84 (77.1%)	121 (72.0%)	
**Tumor T status, *n* (%)**			
T1+T2	59 (54.1%)	107 (63.7%)	0.113
T3+T4	50 (45.9%)	61 (36.3%)	
**Lymph node status, *n* (%)**			
Negative	28 (25.7%)	53 (31.5%)	0.295
Positive	81 (74.3%)	115 (68.5%)	
**Distant metastasis, *n* (%)**			
Negative	53 (48.6%)	80 (47.6%)	0.870
Positive	56 (51.4%)	88 (52.4%)	
**Cell differentiation, *n* (%)**			
Well/Moderately	87 (79.8%)	158 (94.0%)	<0.001
Poorly	22 (20.2%)	10 (6.0%)	

Categorical data: *n* (%); Continuous variables: mean ± standard deviation (SD). Mann–Whitney U-test or Fisher’s exact test was used to evaluate the comparisons between the wild-type and mutation type EGFR in lung adenocarcinoma patients. A *p* value of < 0.05 was defined as statistically significant.

**Table 2:** Distribution frequencies of tissue inhibitor of metalloproteinases (TIMP)-3 genotypes of patients with lung adenocarcinoma and multiple logistic regression analysis of epidermal growth factor receptor (EGFR) mutation associations.

Genotype SNP	Wild-Type (*N* = 109)	Mutation Type (*N* = 168)	AOR (95% CI)	*p*-Value
**rs9619311**				
TT	89 (81.7%)	133 (79.2%)	1.00	
TC	19 (17.4%)	33 (19.6%)	1.228 (0.621–2.428)	0.555
CC	1 (0.9%)	2 (1.2%)	1.229 (0.103–14.708)	0.871
TC+CC	20 (18.3%)	35 (20.8%)	1.228 (0.631–2.390)	0.545
**rs9862**				
CC	43 (39.4%)	49 (29.2%)	1.00	
CT	47 (43.1%)	69 (41.1%)	1.423 (0.778–2.602)	0.252
TT	19 (17.4%)	50 (29.7%)	2.530 (1.230–5.205)	0.012 *
CT+TT	66 (60.6%)	119 (70.8%)	1.748 (1.002–3.048)	0.049 *
**rs11547635**				
CC	52 (47.7%)	85 (50.6%)	1.00	
CT	44 (40.4%)	68 (40.5%)	0.891 (0.514–1.544)	0.680
TT	13 (11.9%)	15 (8.9%)	0.611 (0.253–1.474)	0.273
CT+TT	57 (52.3%)	83 (49.4%)	0.826 (0.492–1.387)	0.469

Abbreviations: SNP—single-nucleotide polymorphism. The adjusted odds ratios (AORs) with their 95% confidence intervals (CIs) were estimated by multiple logistic regression models after controlling for age, gender, and cigarette smoking. * *p* Value is less than 0.05.

**Table 3:** Distribution frequencies of tissue inhibitor of metalloproteinases (TIMP)-3 genotypes of lung adenocarcinoma patients with different cigarette smoking statuses and multiple logistic regression analysis of epidermal growth factor receptor (EGFR) mutation associations.

Genotype SNP	Non-Smoking (*N* = 179)			Smoking (*N* = 98)		
	Wild-Type (*N* = 49)	Mutation Type (*N* = 130)	*p*-Value	Wild-Type (*N* = 60)	Mutation Type (*N* = 38)	*p*-Value
**rs9619311**						
TT	39 (79.6%)	106 (81.5%)		50 (83.3%)	27 (71.1%)	
TC	9 (18.4%)	23 (17.7%)	0.675	10 (16.7%)	10 (26.3%)	0.268
CC	1 (2.0%)	1 (0.8%)	0.423	0 (0.0%)	1 (2.6%)	–
TC+CC	10 (20.4%)	24 (18.5%)	0.556	10 (16.7%)	11 (28.9%)	0.190
**rs9862**						
CC	18 (36.7%)	42 (32.3%)		25 (41.7%)	7 (18.4%)	
CT	22 (44.9%)	50 (38.5%)	0.853	25 (41.7%)	19 (50.0%)	0.169
TT	9 (18.4%)	38 (29.2%)	0.116	10 (16.6%)	12 (31.6%)	0.045 *^,a^
CT+TT	31 (63.3%)	88 (67.7%)	0.386	35 (58.3%)	31 (81.6%)	0.070
**rs11547635**						
CC	25 (51.0%)	62 (47.7%)		27 (45.0%)	23 (60.5%)	
CT	18 (36.7%)	55 (42.3%)	0.736	26 (43.3%)	13 (34.2%)	0.235
TT	6 (12.3%)	13 (10.0%)	0.661	7 (11.7%)	2 (5.3%)	0.317
CT+TT	24 (49.0%)	68 (52.3%)	0.903	33 (55.0%)	15 (39.5%)	0.168

^a^ AOR (95% CI): 3.497 (1.027–11.904). The adjusted odds ratios (AORs) with their 95% confidence intervals (CIs) were estimated by multiple logistic regression models after controlling for age and gender. * *p* Value is less than 0.05.

**Table 4:** Associations between the tissue inhibitor of metalloproteinases (TIMP)-3 genotypes with the epidermal growth factor receptor (EGFR) L858R mutation and exon 19 in-frame deletion in patients with lung adenocarcinoma.

SNP Genotypes	Wild-Type (*N* = 109)	L858R		Exon 19 In-Frame Deletion	
		(*N* = 78)	AOR (95% CI)	(*N* = 81)	AOR (95% CI)
**rs9619311**					
TT	89 (81.7%)	64 (82.1%)	1.00	64 (79.0%)	1.00
TC	19 (17.4%)	13 (16.7%)	0.701 (0.277–1.772)	16 (19.8%)	1.147 (0.514–2.557)
CC	1 (0.9%)	1 (1.2%)	0.560 (0.033–9.449)	1 (1.2%)	1.340 (0.081–22.202)
TC+CC	20 (18.3%)	14 (17.9%)	0.689 (0.280–1.695)	17 (21.0%)	1.157 (0.529–2.534)
**rs9862**					
CC	43 (39.4%)	23 (29.5%)	1.00	23 (28.4%)	1.00
CT	47 (43.1%)	30 (38.5%)	1.343 (0.614–2.938)	37 (45.7%)	1.560 (0.760–3.204)
TT	19 (17.4%)	25 (32.0%)	2.975 (1.182–7.488) ^a^	21 (25.9%)	2.295 (0.973–5.412)
CT+TT	66 (60.6%)	55 (70.5%)	1.787 (0.873–3.661)	58 (71.6%)	1.772 (0.908–3.458)
**rs11547635**					
CC	52 (47.7%)	40 (51.3%)	1.00	39 (48.1%)	1.00
CT	44 (40.4%)	32 (41.0%)	1.005 (0.493–2.046)	33 (40.7%)	0.932 (0.486–1.787)
TT	13 (11.9%)	6 (7.7%)	0.536 (0.166–1.731)	9 (11.2%)	0.885 (0.325–2.410)
CT+TT	57 (52.3%)	38 (48.7%)	0.883 (0.453–1.722)	42 (51.9%)	0.922 (0.499–1.702)

SNP—single-nucleotide polymorphism. The adjusted odds ratios (AORs) with their 95% confidence intervals (CIs) were estimated by multiple logistic regression models after controlling for age, gender, and cigarette smoking. ^a^ *p* = 0.021.

**Table 5:** Clinicopathologic characteristics of lung adenocarcinoma patients, stratified by polymorphic genotypes of tissue inhibitor of metalloproteinases (TIMP)-3 rs9862.

Variable	All (*N* = 277)			Males (*N* = 125)			Females (*N* = 152)		
	CC (*N* = 92)	CT+TT (*N* = 185)	*p*-Value	CC (*N* = 39)	CT+TT (*N* = 86)	*p*-Value	CC (*N* = 53)	CT+TT (*N* = 99)	*p*-Value
**Stage**									
I+II	26 (28.3%)	46 (24.9%)	0.544	13 (33.3%)	14 (16.3%)	0.032 *^,a^	13 (24.5%)	32 (32.3%)	0.316
III+IV	66 (71.7%)	107 (75.1%)		26 (66.7%)	72 (83.7%)		40 (75.5%)	67 (67.7%)	
**Tumor T status**									
T1+T2	58 (63.0%)	108 (58.4%)	0.456	24 (61.5%)	47 (54.7%)	0.471	34 (64.2%)	61 (61.6%)	0.758
T3+T4	34 (37.0%)	77 (41.6%)		15 (38.5%)	39 (45.3%)		19 (35.8%)	38 (38.4%)	
**Lymph node status**									
Negative	29 (31.5%)	52 (28.1%)	0.556	10 (25.6%)	19 (22.1%)	0.663	19 (35.8%)	33 (33.3%)	0.755
Positive	63 (68.5%)	133 (71.9%)		29 (74.4%)	67 (77.9%)		34 (64.2%)	66 (66.7%)	
**Distant metastasis**									
Negative	42 (45.7%)	91 (49.2%)	0.579	19 (48.7%)	38 (44.2%)	0.637	23 (43.4%)	53 (53.5%)	0.233
Positive	50 (54.3%)	94 (50.8%)		20 (51.3%)	48 (55.8%)		30 (56.6%)	46 (46.5%)	
**Cell differentiation**									
Well/Moderately	84 (91.3%)	161 (87.0%)	0.294	33 (84.6%)	69 (80.2%)	0.558	51 (96.2%)	92 (92.9%)	0.412
Poorly	8 (8.7%)	24 (13.0%)		6 (15.4%)	17 (19.8%)		2 (3.8%)	7 (7.1%)	

^a^ Odds ratio (OR) (95% confidence interval (CI)): 2.571 (1.069–6.187). * *p* Value is less than 0.05.

**Table 6:** Clinicopathologic characteristics of male lung adenocarcinoma patients with the epidermal growth factor receptor (EGFR) mutation, stratified by polymorphic genotypes of tissue inhibitor of metalloproteinases (TIMP)-3 rs9862.

Variable	Wild-type (*N* = 65)			Mutation type (*N* = 60)		
	CC (*N* = 24)	CT+TT (*N* = 41)	*p*-Value	CC (*N* = 15)	CT+TT (*N* = 45)	*p*-Value
**Stage**						
I+II	6 (25.0%)	6 (14.6%)	0.299	7 (46.7%)	8 (17.8%)	0.025 *^,a^
III+IV	18 (75.0%)	35 (85.4%)		8 (53.3%)	37 (82.2%)	
**Tumor T status**						
T1+T2	15 (62.5%)	21 (51.2%)	0.377	9 (60.0%)	15 (57.8%)	0.880
T3+T4	9 (37.5%)	20 (48.8%)		6 (40.0%)	19 (42.2%)	
**Lymph node status**						
Negative	3 (12.5%)	10 (24.4%)	0.247	7 (46.7%)	9 (20.0%)	0.043 *^,b^
Positive	21 (87.5%)	31 (75.6%)		8 (53.3%)	36 (80.0%)	
**Distant metastasis**						
Negative	11 (45.8%)	20 (48.8%)	0.818	8 (53.3%)	18 (40.0%)	0.367
Positive	13 (54.2%)	21 (51.2%)		7 (46.7%)	27 (60.0%)	
**Cell differentiation**						
Well/Moderately	19 (79.2%)	30 (73.2%)	0.588	14 (93.3%)	39 (86.7%)	0.486
Poorly	5 (20.8%)	11 (26.8%)		1 (6.7%)	6 (13.3%)	

^a^ Odds ratio (OR) (95% confidence interval (CI)): 4.047 (1.136–14.411). ^b^ OR (95% CI): 3.500 (1.003–12.216). * *p* Value is less than 0.05.

## Abbreviations

AORs	Adjusted odds ratios
CIs	Confidence intervals
EGFR	Epidermal growth factor receptor
GTEx	Genotype-Tissue Expression
LADC	Lung adenocarcinoma
MMP	Matrix metalloproteinase
NSCLC	Non-small-cell lung cancer
SCC	Squamous cell carcinoma
SNPs	Single-nucleotide polymorphisms
TCGA	The Cancer Genome Atlas
